# Spatiotemporally comparative analysis of three common infectious diseases in China during 2013–2015

**DOI:** 10.1186/s12879-022-07779-4

**Published:** 2022-10-18

**Authors:** Yang Shao, Meifang Li, Jin Luo, Le Yu, Xia Li

**Affiliations:** 1 School of Geography and Environment, Jiangxi Normal University, Nanchang, 330022 Jiangxi People’s Republic of China; 2 School of Geography and Planning, Sun Yat-Sen University, Guangzhou, 510006 Guangdong Province People’s Republic of China; 3 Department of Geography, Dartmouth College, Hanover, NH 03755 USA; 4 Nanchang City Center for Disease Control and Prevention, Nanchang, 330038 Jiangxi People’s Republic of China; 5 Department of Earth System Science, Ministry of Education Key Laboratory for Earth System Modeling, Institute for Global Change Studies, Tsinghua University, Beijing, 100084 China; 6 Ministry of Education Ecological Field Station for East Asian Migratory Birds, Beijing, 100084 China; 7 School of Geographic Sciences, Key Lab of Geographic Information Science (Ministry of Education), East China Normal University, Shanghai, 200241 China

**Keywords:** Dengue fever, Influenza, Hand, foot, and mouth disease, Spatiotemporal distribution, Spatial scan statistic, Geographic Information Science

## Abstract

**Background:**

Dengue fever (DF), influenza, and hand, foot, and mouth disease (HFMD) have had several various degrees of outbreaks in China since the 1900s, posing a serious threat to public health. Previous studies have found that these infectious diseases were often prevalent in the same areas and during the same periods in China.

**Methods:**

This study combined traditional descriptive statistics and spatial scan statistic methods to analyze the spatiotemporal features of the epidemics of DF, influenza, and HFMD during 2013–2015 in mainland China at the provincial level.

**Results:**

DF got an intensive outbreak in 2014, while influenza and HFMD were stable from 2013 to 2015. DF mostly occurred during August–November, influenza appeared during November–next March, and HFMD happened during April–November. The peaks of these diseases form a year-round sequence; Spatially, HFMD generally has a much higher incidence than influenza and DF and covers larger high-risk areas. The hotspots of influenza tend to move from North China to the southeast coast. The southeastern coastal regions are the high-incidence areas and the most significant hotspots of all three diseases.

**Conclusions:**

This study suggested that the three diseases can form a year-round sequence in southern China, and the southeast coast of China is a particularly high-risk area for these diseases. These findings may have important implications for the local public health agency to allocate the prevention and control resources.

## Background

Dengue fever (DF), influenza, and hand, foot, and mouth disease (HFMD) are listed as notifiable infectious diseases in China [1–3], due to their prevalence and the great public health burden they have caused to society. DF is an acute infectious disease caused by the dengue virus, mainly transmitted by mosquitos and found in more than 100 countries and regions worldwide, covering nearly one-third of the global population [4, 5]. In China, DF first appeared in 1873 [6], and the largest outbreak with 48,162 cases nationwide occurred in 2014 [7, 8]. Influenza, one of the most common infectious diseases, is mainly caused by influenza A, B, and C viruses. These viruses not only have strong infectivity but also a fast mutation rate, posing a serious threat to public health [9]. HFMD is another common infectious disease caused by a variety of enteroviruses, and mainly affects infants and young children. HFMD has had several large-scale outbreaks in China in recent years, with many serious or even death cases [10–12].

Previous studies have studied the spatial and temporal characteristics of the three diseases. For example, Wu et al. [13] investigated foreigner DF patients in China during 2005–2017. They found that most of them were imported from Myanmar, and the foreigners in Yunnan Province are markedly different from that in other provinces of China [13]; Dhewantara et al. [14] found that during 2007–2013 the DF epidemic had an obvious annual spreading process from the city center of Cima City, Indonesia to surrounding areas, and the cases were concentrated in the spring season. Liu et al. [15] mapped the incidence of seasonal influenza in Gansu Province, China, and found that influenza epidemics among eight climate zones form significant spatial clusters, and the southeastern part of Gansu Province characterized by dry winter and warm summer was the high-risk area; Yang et al. [16] found that during 2017–2018, influenza had a considerably higher incidence in the winter and spring seasons. Song et al. [17] found significant positive global spatial autocorrelation in the HFMD epidemics during 2016–2018 in Inner Mongolia, China, and “high–high” clusters are most common in the 2017 epidemic; Wang et al. [18] found a potential seasonality in the HFMD epidemics in China from May 2008 to November 2012.

Most studies focused on a single disease, and the comparative analysis that considers more than one disease at the same time is rare in the literature. However, epidemics of different kinds of diseases may have considerable spatiotemporal overlaps, motivating comparative analyses of different diseases and aiming to optimization of public health resource allocation. A notable example is the occurrence of a huge DF outbreak, influenza outbreaks, and HFMD outbreaks in Guangdong Province, China in 2014 [19]. Studies have found that all three diseases are highly seasonal, regional, and climate-sensitive [20–22]. For instance, Li et al. [20] found that DF was significantly associated with both temperature and precipitation, and the effect of temperature on DF was most pronounced in high-income subtropical areas; Zhao et al. [22] suggested that the spatial transmission of influenza A (H1N1) was associated with temperature, sunlight hours, precipitation, atmospheric pressure, absolute and relative humidity, and was largely driven by the absolute humidity; Wu et al. [23] found that the HFMD occurrences showed significant seasonality, with the rainfall, temperature, and humidity as the significant risk factors and wind speed as a protective factor. Due to the similarity and comparability among DF, influenza, and HFMD epidemics, a comparative analysis can provide scientific evidence for guiding the optimal allocation of the prevention and treatment resources, especially when these resources are limited.

Using the epidemic data of DF, influenza, and HFMD during 2013–2015 in mainland China, this study combined descriptive statistical analysis and spatial scan statistics to explore the characteristics of their spatiotemporal distributions, as well as the similarities and differences among them. The results of this study not only can provide basic knowledge about the spatiotemporal distributions of the three diseases in China, but can also inform intervention planning and policymaking.

## Methods

### Data description

We obtained the epidemic information of DF, influenza, and HFMD in China during 2013–2015 from the National Population Health Data Center of the Chinese Center for Disease Control Prevention [24]. We chose this time period of DF, influenza, and HFMD epidemics for studying, on the one hand, is limited by the data we obtained, on the other hand, there was an outbreak of DF in 2014, which makes it comparable with the other two diseases. The epidemic information includes the monthly case count and monthly crude incidence at the provincial level for each of the three diseases. The provincial monthly crude incidence was calculated with Eq. (1):

1 $$Provincial\, monthly \,crude \,incidence =\frac{the\, number\, of\, cases\, for\, the \,province\, in\, the\, month}{the \,number\, of\, popultion\, for\, the\, province\, in\, that\, year}$$

With the information on the case count and crude incidence, we inferred the population of each province for each year during 2013–2015, which was used as the population background for cluster detection analysis.

The provincial geographic data was acquired from the National Geomatics Center of China [25].

### Descriptive statistical analysis

With descriptive statistical analysis and GIS visualization tools, this study analyzed the monthly distributions of each of three epidemics during 2013–2015 and mapped the spatial distribution of annual incidence of each of three epidemics across 31 provinces in mainland China [26–28].

### Spatial scan statistics analysis

The spatial scan statistics analysis was employed to detect the spatial clusters of the yearly provincial incidence of DF, influenza, and HFMD in mainland China during 2013–2015. The essence of the spatial scan statistics analysis is to use the maximum likelihood principle to locate the strongest cluster in the study area [29–31]. The working process is that: it uses a circular or elliptical window to scan every location in the study area. For each location, the radius of the scanning window continuously changes from zero to the upper limit defined by the user [32]. For the scanning window of any size, the log-likelihood ratio (LLR) was calculated based on the data of disease and population within and outside that window. The clusters are ranked by the LLR value and their statistical significance is evaluated with Monte Carlo simulations. The window with the largest LLR value is deemed as the first cluster (or primary cluster) when the P-value is significant, i.e., less than 0.05, and the other significant windows are considered the secondary clusters [27, 33]. Meanwhile, the Observed/Expected (ODE) and Relative Risk (RR) are used to quantify the risk of clusters. ODE is calculated as the observed cases within the cluster divided by the expected cases within the cluster, in other words, the estimated risk within the cluster is divided by the estimated risk for the study area as a whole. RR is the estimated risk within the cluster divided by the estimated risk outside the cluster. It is calculated as the observed incident number divided by the expected incident number within the cluster divided by the observed incident number divided by the expected incident number outside the cluster.

In this study, we used this method to detect those provinces with abnormally high risks for the three diseases, and descriptively compared the high-risk areas of the three diseases. When running the cluster analysis, the centroid point of each province was used to represent the province. We set the maximum cluster size to be 50% of the population at risk and the number of Monte Carlo iterations for evaluating statistical significance to be 999 [34, 35]. The SaTScan 9.5 was used to implement the spatial scan statistics analysis [36], and the outputs were presented with ArcGIS 10.2 [37].

## Results

### Temporal distributions of DF, influenza, and HFMD epidemics

**Fig. 1:**
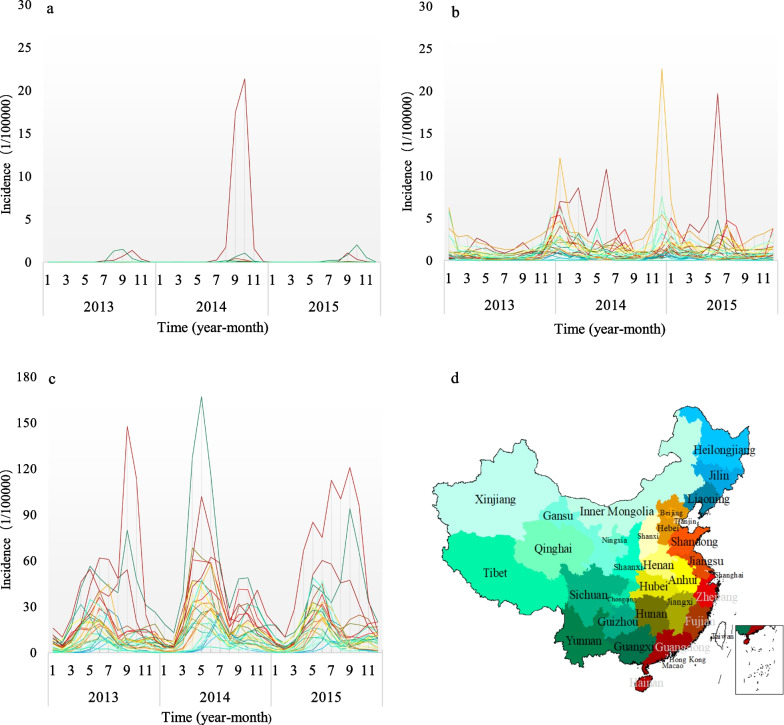
The monthly incidence of DF, influenza, and HFMD epidemics in mainland China, 2013–2015. **a**–**c** Represent the monthly incidence of DF, influenza, and HFMD epidemics, respectively. Each line in each panels represents a province, and its color is consistent with the fill color in **d**

Figure 1 shows the monthly incidences of three diseases in 31 provinces during 2013–2015: across the entire year, the incidence of HFMD is much higher than that of influenza and DF, with DF being the lowest among the three; the peaks of DF and HFMD overlapped between August to November, whereas their valleys occurred between December and March of the following year; those eastern coastal provinces have higher incidences (the red lines in the charts) of all three diseases than the western and northeastern regions (the blue-green lines in the charts). The period of the high incidence of DF is mainly August to November, and the peak is in October, while the low incidence appeared between December and July of the following year. The south coastal provinces all have a high incidence of DF, and the highest incidence occurred in October 2014; The period of the high incidence of influenza is generally from November to March of the following year, which is consistent with the high incidence in autumn and winter as influenza viruses are not heat-resistant, while southern provinces also experienced high incidence between May and August in 2014 and 2015. The provinces and cities with a high incidence of influenza are mainly south coastal provinces and north inland cities, and the highest incidence of influenza appeared in December 2014; Fig. 1c reflects that the period of the high incidence of HFMD is located in April–November and with a low incidence from December to March next year. The southeast coastal provinces all have a high HFMD incidence, with the highest incidence occurring in May 2014.

**Fig. 2:**
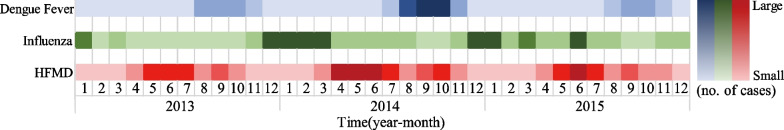
Number of cases of DF, influenza, and HFMD in mainland China during 2013–2015

The HFMD incidence in various areas changed more obviously from 2013 to 2015, and was generally higher than that of DF and influenza in the same period. Figure 2 illustrated the peak periods of these three kinds of epidemics during 2013–2015 were continuous and formed a 1-year circle. Generally, it started with influenza epidemics whose peak period is from December to next March, then followed by HFMD epidemics whose peak period is from April to July, and the last is the DF epidemics whose peak period is from August to November. However, as the HFMD case count far exceeded that of DF and influenza, its prevalent periods were relatively longer, e.g., April–November.

### Spatial distributions of DF, influenza, and HFMD epidemics

**Fig. 3:**
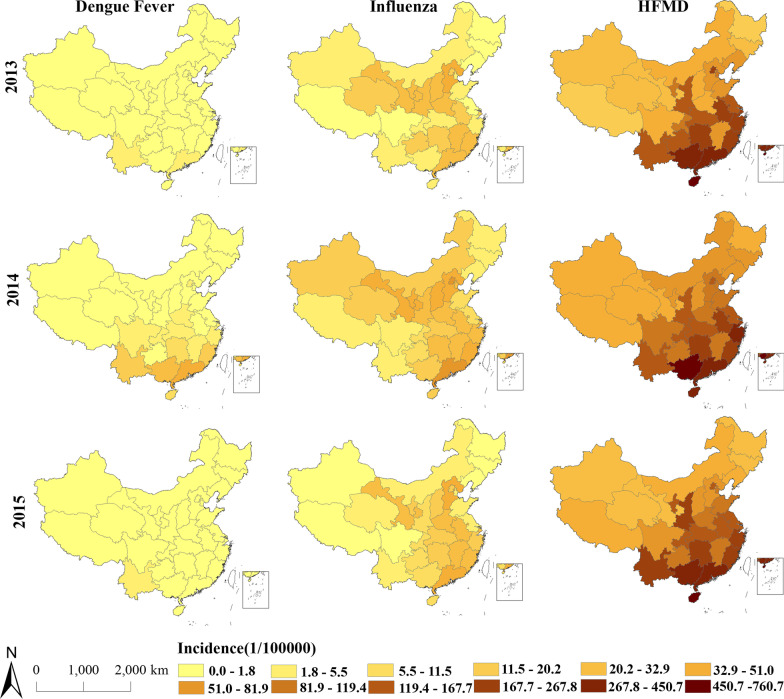
Spatial distribution of yearly incidence of DF, influenza, and HFMD in mainland China, 2013–2015

Figure 3 maps the yearly incidence of the three diseases across mainland China during 2013–2015. As shown in Fig. 3, HFMD has the darkest hue on the map, that is, it has a generally higher incidence, and the lowest incidence is generally of DF; the spatial distribution of the incidence of HFMD has not changed significantly between 2013 and 2015, while the tone of DF and influenza significantly deepened in some provinces in 2014; the areas with relatively high incidence for each of the three diseases were mostly located in the southeast coastal provinces. As a longitudinal comparison among the spatial distributions of the incidence of three common infectious diseases over 3 years: the higher DF incidence was mainly concentrated in the south and southwest provinces, and the highest incidence of DF occurred in 2014, while in 2015, there was a significant decrease; the influenza incidence was generally higher in 2014, and the higher incidence was located in the central and southeastern regions, extending from the center to the northwestern regions; the HFMD incidence was relatively similar among 3 years, suggesting that the annual variation of the HFMD prevalence was small.

On the whole, HFMD has the highest incidence and the annual difference is small; unlike DF and HFMD, which have a higher incidence in southern China, the areas with a high incidence of influenza extend to northwest China; the incidence of DF was significantly lower than that of influenza and HFMD, and showed significant annual differences.

### Spatial clusters of DF, influenza, and HFMD epidemics

#### Number of significant clusters of DF, influenza, and HFMD epidemics

The numbers of significant clusters of three common infectious diseases from 2013 to 2015 in mainland China were listed in Table 1: the number of HFMD significant clusters is the most, while the least number of significant clusters is DF; the minimum number of significant clusters of DF and HFMD both appeared in 2014, and the number of clusters showed a trend of decreasing firstly and then increasing during the 3 years, while the number of significant clusters of influenza was relatively stable; the minimum number of significant clusters was DF which has only one cluster in 2014, and the maximum number was HFMD in 2015. Comparing the number of significant clusters of the same disease in different years, we can find that: the number of DF significant clusters was the same in 2013 and 2015, while there was only one in 2014; the number of influenza significant clusters in 2013 was the same as that in 2014, and there was a slight decrease in 2015; the number of HFMD significant clusters varied over the 3 years, with the least in 2014 and the most in 2015.

**Table 1:** The number of significant clusters of DF, influenza, and HFMD epidemics in mainland China, 2013–2015

Disease	Year	Number of significant clusters
DF	2013	3
	2014	1
	2015	3
Influenza	2013	8
	2014	8
	2015	7
HFMD	2013	10
	2014	8
	2015	11

In summary, the number of DF significant clusters in mainland China between 2013 and 2015 was relatively small, with a maximum of three, while the number of significant clusters of influenza and HFMD was seven or more, indicating that there were fewer regions with spatially significant hotspots in the DF epidemics; on the other hand, influenza and HFMD have formed spatial hot spots in many parts of the country, suggesting that the outbreaks of influenza and HFMD have a higher risk in more areas.

#### First spatial clusters of DF, influenza, and HFMD epidemics

**Fig. 4:**
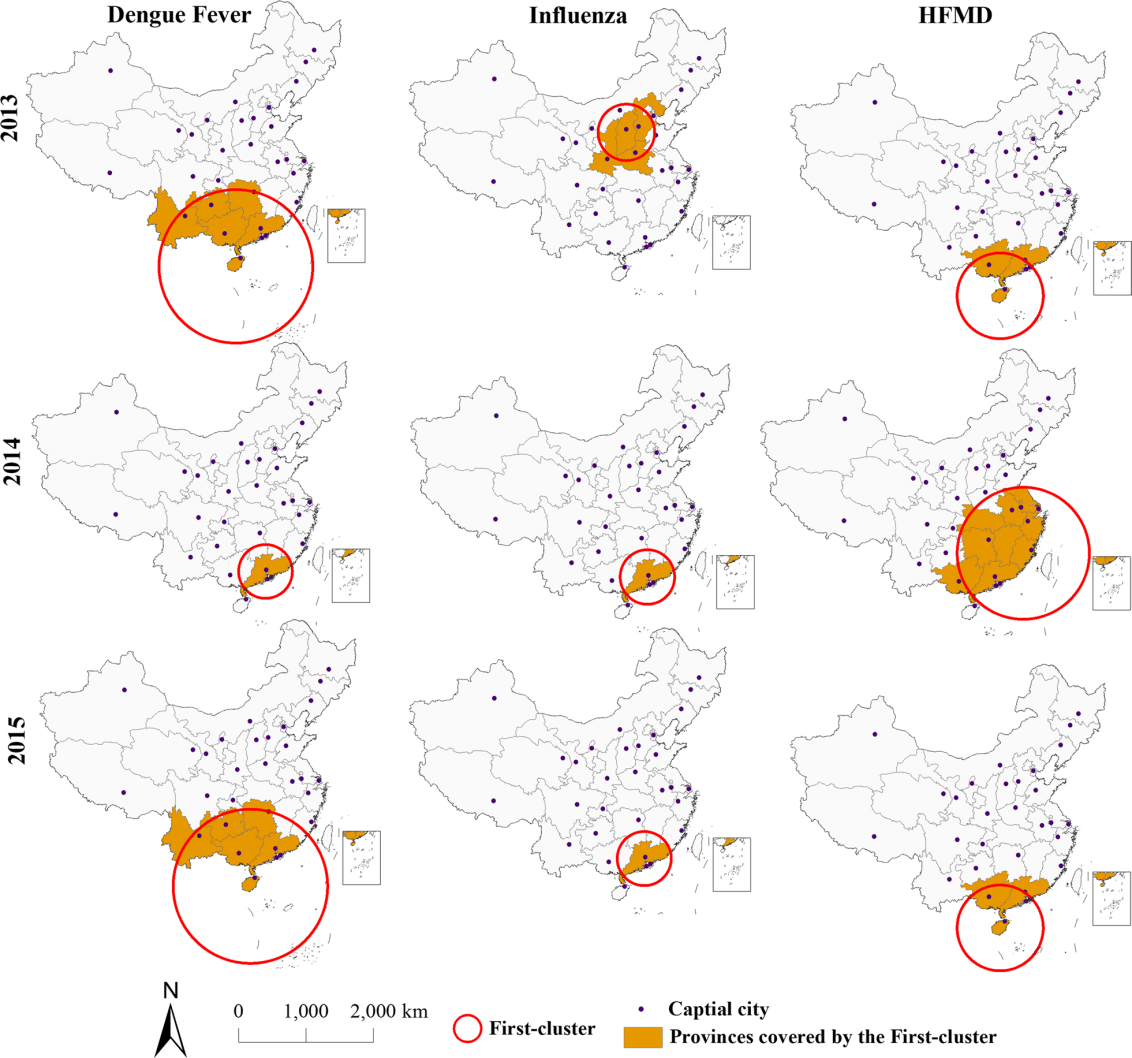
Spatial distribution of the first cluster of DF, influenza, and HFMD epidemics in mainland China, 2013–2015

Figure 4 maps of the first cluster reported by SaTScan for each of the three diseases (marked with the red circle). In these maps, the provincial units colored with orange are the areas being part of the first cluster. Table 2 lists the attributes of the first clusters mapped in Fig. 4. The value of the radius indicates the coverage of the circular cluster. When the radius of the cluster is 0 km, it represents that the first cluster only covers a separate administrative division (province or city in this study).

**Table 2:** Characteristics of the first cluster of DF, influenza, and HFMD epidemics in mainland China, 2013–2015

Disease	Year	LLR	P-value	ODE	RR	Radius (km)
DF	2013	5725.66*	< 0.0001	4.15	69.09*	1064.53
	2014	107,309.46*	< 0.0001	12.12	312.20*	0
	2015	4165.46*	< 0.0001	3.98	36.51*	1064.53
Influenza	2013	10,862.14*	< 0.0001	1.96	2.48*	427.52
	2014	25,004.67*	< 0.0001	3.00	3.62*	0
	2015	22,763.13*	< 0.0001	3.00	3.62*	0
HFMD	2013	297,263.99*	< 0.0001	2.82	3.76*	597.29
	2014	308,556.12*	< 0.0001	1.55	2.62*	958.01
	2015	283,609.24*	< 0.0001	2.69	3.49*	597.29

Conducted using Discrete Poisson Model. *P < 0.05

As shown in Fig. 4 and Table 2, the first cluster of DF and HFMD covered more provinces and cities over the 3 years, with the least amount of influenza, which is reflected by the radius of the first cluster of DF and HFMD is larger than that of influenza. The center of the first cluster of DF in 2014 is the same as that of influenza in 2014 and 2015, covering Guangdong province only (surrounded by the red circle). The highest intensity of hot spots of DF was recorded in 2014, with values of LLR, ODE, and RR being the maximum; the provinces covered by the first cluster of DF were the same as that of HFMD in 2013 and 2015, but with a slight difference in intensity, that is, the intensity of the first cluster of DF in 2013 was slightly higher than that in 2015, while the intensity of the first cluster of HFMD was similar in these 2 years.

By comparing the spatial distribution and attribute characteristics of the first cluster of each disease in 3 years, it can be found that: the first cluster of DF in 2013 and 2015 covered the same provinces and cities, and centers, that is, the radius of the first cluster was the same. The coverage of the first cluster of DF was the smallest in 2014 in Guangdong province, but with the most significant and intense clustering, as shown in the maximum values of LLR, ODE, and RR, as well as a significant decrease in 2015 compared with the risk of the first cluster of DF in 2014 (values of ODE and RR decreased significantly), indicating that the government’s prevention and control measures effectively reduced the prevalent level of disease after the outbreak of DF in 2014; from 2014 to 2015, the first cluster of influenza covers the same provinces and cities, the risk level of hot spots of disease is the same (values of ODE and RR are the same), and the clustering and intensity of the first cluster are similar. The center of the first cluster of influenza shifted from the north to the southeastern coastal region with the increasing disease risk and intensity of the first cluster from 2013 to 2014; in 2014, the first cluster of HFMD covered most provinces and cities and had the most significant clustering, which is reflected in the maximum value of LLR and radius of the first cluster. In terms of the risk of these diseases, all the highest risks occurred in 2014, with the maximum values of ODE and RR in 3 years, but HFMD had similar intensity in the first clusters of all 3 years, and there was no significant change in the risk of disease.

This analysis revealed that most of the first clusters are located in the south and southeast coastal regions, while the changes in the first cluster and coverage often reflect the prevalence of disease and the emergence of high-risk areas, with the greatest change occurring in the first cluster of influenza, showing a trend of shifting from the north to the southeast coast. In a word, provinces among the southeast coastal regions are not only a high epidemic area, but also have significant and high-intensity spatial clustering. The outbreak of DF was more severe, with a wider epidemic range and higher intensity of spatial clustering, but the significant reduction in the risk of DF in 2015 also suggested that the effect for prevention and control was the best.

## Discussion

DF, influenza, and HFMD are common infectious diseases in mainland China. In this study, we depicted and compared their temporal and spatial distributions in mainland China using the provincial-level monthly incidence data of 2013–2015. Further, we used the spatial scan statistic method to detect spatial clusters of each of the three diseases based on the provincial-level yearly incidence data and compared the clusters among the three diseases.

In terms of temporal characteristics, we found that the high incidence of DF occurred between August and November, which is the period of rapid breeding of the *Aedes aegypti* mosquito, the most important vector of dengue fever virus [20, 38, 39]. At the same time, the periods of HFMD prevalence were April–November, thus, the high incidence of DF and HFMD had a yearly overlap during August–November. On the other hand, the prevalent period of influenza was mainly from November to March (late autumn and winter), just the opposite of that of HFMD (spring and summer). This seasonality of the two diseases is mainly due to influenza viruses being heat-intolerant, whereas high temperatures are conducive to the propagation of HFMD viruses. Our findings are consistent with previous studies on the prevalence period of the three diseases in China [8, 16, 40]. Moreover, we can see that these diseases can form a year-round sequence that may have important implications for the local public health agency.

In terms of the spatial distribution of the monthly incidence, we found that (1) the high-incidence areas of all three diseases are mainly located in the southeastern coastal regions; (2) HFMD, which generally has a much higher incidence than the other two diseases, also covers a larger high-risk area; (3) after the outbreak in 2014, the epidemic region of DF considerably shrunk in 2015, whereas influenza and HFMD remained to have a great impact on most areas of mainland China.

Through the cluster detection with the spatial scan statistics method, we found that (1) each of the three diseases has abnormally high-risk areas in mainland China; (2) the first clusters of three common diseases in the three study years are mostly located in the southern and southeastern coastal areas, and in 2014–2015, their first clusters had an overlap in south coastal areas; (3) the first cluster of influenza shows the most drastic dynamic during the 3 years, moving from North China to the southeast coast. This last finding is new, because previous studies of clusters of influenza in China have been either about a local region or about one time period [15, 41, 42], and therefore not able to identify the dynamic on a national scale. The clusters of DF we identified are mostly related to the outbreaks in Guangdong province in 2014, which confirmed the findings of previous studies [43, 44]. Compared with influenza and DF, HFMD has a much larger number of significant clusters and those clusters cover a much more extensive area.

## Conclusions

Through the comparative analysis of the spatiotemporal distributions of DF, influenza, and HFMD epidemics in mainland China during 2013–2015, we got several hints for the prevention and control of these three common infectious diseases: Firstly, we should take precautions against HFMD during spring and summer seasons, pay more attention to DF and HFMD in late summer and autumn seasons, and beware of influenza during late autumn and winter, which forms a year-round sequence. Secondly, the south and southeast coastal areas of China are at high risk of all these diseases, which should be given more attention, while the northern regions also need to be prevented the prevalence of influenza. Thirdly, when the resources for prevention and control of infectious diseases are limited and restricted in reality, this study suggests that the prevention and control of these three common infectious diseases need a tendency to allocate resources for prevention and control. For example, the national government should direct the resources for prevention and control toward south coastal provinces, and because of the high incidence of influenza and HFMD keeping for a long time, it is necessary to maintain the long-term focus on prevention [45, 46].

Finally, the study found that the first cluster of influenza showed a trend of shifting from the north to the southeastern coastal region, which may be due to regional differences in climatic factors or social factors such as human and economic factors, and it is worthy of further studying. In addition, due to the availability of data, there are certain limitations in this paper, such as the fact that most of the indicators presented in this paper are crude incidence data, which do not take into account the differences in population movement [47] and demographic structure between regions; in terms of research scale, the temporal scale of this paper is as fine as a month, and the spatial scale is provincial and municipal administrative units, which fail to explore the patterns and differences of disease prevalence on a finer spatiotemporal scale.

## Data Availability

All data generated or analysed during this study are included in this published article [and its additional information files].

## Abbreviations

DF: Dengue fever
HFMD: Hand, foot, and mouth disease
LLR: Log-likelihood ratio
ODE: Observed/expected
RR: Relative risk
